# Screening of Miners and Millers at Decreasing Levels of Asbestos Exposure: Comparison of Chest Radiography and Thin-Section Computed Tomography

**DOI:** 10.1371/journal.pone.0118585

**Published:** 2015-03-19

**Authors:** Mario Terra-Filho, Ericson Bagatin, Luiz Eduardo Nery, Lara Maris Nápolis, José Alberto Neder, Gustavo de Souza Portes Meirelles, C. Isabela Silva, Nestor L. Muller

**Affiliations:** 1 Pulmonary Division, Heart Institute, Hospital das Clínicas da Faculdade de Medicina da Universidade de São Paulo, São Paulo, Brazil; 2 Occupational Health Area, Department of Social and Preventive Medicine, Faculdade de Ciências Médicas, Universidade Estadual de Campinas, Campinas, São Paulo, Brazil; 3 Department of Clinical Medicine, Faculdade de Medicina de Jundiaí, Jundiaí, São Paulo, Brazil; 4 Respiratory Division, Department of Medicine, Escola Paulista de Medicina, Universidade Federal de São Paulo, São Paulo, Brazil; 5 Radiology Division, Department of Radiology, Escola Paulista de Medicina, Universidade Federal de São Paulo, São Paulo, Brazil; 6 Radiology Division, Fleury Group, São Paulo, Brazil; 7 Department of Radiology, Delfin Clinic and Portuguese Hospital, Salvador, Bahia, Brazil; 8 Department of Radiology, University of British Columbia, Vancouver, B.C., Canada; University of Montana, UNITED STATES

## Abstract

**Background:**

Chest radiography (CXR) is inferior to Thin-section computed tomography in the detection of asbestos related interstitial and pleural abnormalities. It remains unclear, however, whether these limitations are large enough to impair CXR´s ability in detecting the expected reduction in the frequency of these asbestos-related abnormalities (ARA) as exposure decreases.

**Methods:**

Clinical evaluation, CXR, Thin-section CT and spirometry were obtained in 1418 miners and millers who were exposed to progressively lower airborne concentrations of asbestos. They were separated into four groups according to the type, period and measurements of exposure and/or procedures for controlling exposure: Group I (1940–1966/tremolite and chrysotile, without measurements of exposure and procedures for controlling exposure); Group II (1967–1976/chrysotile only, without measurements of exposure and procedures for controlling exposure); Group III (1977–1980/chrysotile only, initiated measurements of exposure and procedures for controlling exposure) and Group IV (after 1981/chrysotile only, implemented measurements of exposure and a comprehensive procedures for controlling exposure).

**Results:**

In all groups, CXR suggested more frequently interstitial abnormalities and less frequently pleural plaques than observed on Thin-section CT (p<0.050). The odds for asbestosis in groups of decreasing exposure diminished to greater extent at Thin-section CT than on CXR. Lung function was reduced in subjects who had pleural plaques evident only on Thin-section CT (p<0.050). In a longitudinal evaluation of 301 subjects without interstitial and pleural abnormalities on CXR and Thin-section CT in a previous evaluation, only Thin-section CT indicated that these ARA reduced as exposure decreased.

**Conclusions:**

CXR compared to Thin-section CT was associated with false-positives for interstitial abnormalities and false-negatives for pleural plaques, regardless of the intensity of asbestos exposure. Also, CXR led to a substantial misinformation of the effects of the progressively lower asbestos concentrations in the occurrence of asbestos-related diseases in miners and millers.

## Introduction

Asbestos exposure is associated with a number of potential health hazards, including pulmonary fibrosis (asbestosis) and pleural plaques. Intensity of exposure and latency are two major determinants of the risk of disease [1, 2]. Periodic health surveillance of asbestos-exposed subjects therefore should continue for an undetermined period of time even after the cessation of exposure [3, 4].

In this context, chest radiography (CXR) is still widely used to assess the presence and extension of pulmonary and pleural abnormalities in these subjects [5–7]. Thin-section computed tomography, however, has proved to be substantially more sensitive than CXR [8–11] as it may detect incipient interstitial abnormalities (IA) and pleural plaques that are frequently missed at CRX [12, 13]. In a population with pretest risk of exposure to asbestos (i.e. mining workers), several authors have described that the diagnosis or exclusion of asbestos related interstitial fibrosis or pleural plaques, should not be based exclusively on the radiograph, being necessary to confirm these findings on Thin-section CT [1, 2, 9, 12]. These advantages of Thin-section CT are expected to be particularly relevant in subjects with lower pre-test risk of disease and/or those with subtle abnormalities. There is strong evidence that currently asbestos-induced lung diseases are characterized by milder fibrosis than described in classic studies three or more decades ago, a likely consequence of the improvements in exposure control [14–16]. Unfortunately, however, Thin-section CT is a costlier and less-available procedure which is associated with higher radiation exposure [17] compared to CXR. In addition, the higher sensitivity of Thin-section CT may result in an unacceptable rate of incidental findings, leading to further unnecessary evaluations [17–19]. On the other hand, studies of several lung diseases suggest that implementation of low-dose CT scans for screening of early pleuro-pulmonary abnormalities would be economically efficient [20, 21]. Several reasons should be outlined to indicate Thin-section CT in the evaluation of asbestos related diseases: (i) In clinical practice, it is possible to identify the superiority of Thin-section CT compared to the CXR in the identification or exclusion of pleuro pulmonary abnormalities related to exposition, including lung cancer. (ii) workers and employees would be advised to interrupt the exposition by changing their activity in the workplace; and finally (iii) for medico-legal reasons, in order to compensate only workers with high probability of abnormalities related to asbestos exposition [9, 10, 17, 18]. Therefore, a more precise characterization of the actual advantages of Thin-section CT over CXR in epidemiological studies is needed to evaluate its potential role in periodical surveillance of subjects with different pre-test risk of disease, i.e., subjects with different levels of exposure.

The objective of the present study, therefore, was firstly to compare the diagnostic performance of CRX and Thin-section CT in a large group of miners and millers who were separated in groups of decreasing asbestos exposure. In addition, in a longitudinal analysis we investigated the incidence of interstitial and pleural abnormalities on CXR and Thin-section CT in a subgroup of subjects with no previous abnormalities on both methods. Secondarily, we assess whether individuals exposed to asbestos with and without interstitial and pleural changes have differences in spirometric variables.

We hypothesized that the high rates of pleuro-pulmonary abnormalities would impair the ability of CXR to adequately characterize the expected reduction in the risk of disease as exposure decreased.

## Methods

### Study population

This is a cross-sectional study [22] of a population of 1418 workers and ex-workers of a single asbestos mining and milling company (SAMA S.A—Minerações Associadas), who underwent CRX, Thin-section CT and spirometry and were part of larger group which had been previously evaluated [14]. This population was categorized into four groups based on the historical developments of asbestos mining and milling in Brazil: (a) Exposed from 1940 to 1966 in the São Félix mine (State of Bahia, Northeast Region), where measurements of exposure and/or procedures for controlling exposure were not operational and tremolite was found in association with chrysotile (Group I, n = 123); (b) Exposed from 1967 to 1976 in the Canabrava mine (State of Goiás, Central Region) where despite no evidence of amphyboles contamination there were no measurements of exposure and/or procedures for controlling exposure (Group II, n = 600), (c) from 1977 to 1980 when routine airborne fibre measurements were initiated and a program for controlling exposure was progressively set in the Canabrava mine (Group III, n = 479) and (d) from 1981 onwards when, in addition to airborne fibre measurements, a comprehensive controlled-exposure program was fully operational (Group IV, n = 216). A subgroup of subjects without any Thin-section CT and CXR abnormalities at baseline had been examined at two different time points with CXR and Thin-section CT (N = 301). In these subjects, we performed a longitudinal analysis to assess the incidence of pulmonary disease (asbestosis) and pleural disease (pleural plaques). Informed consent for participation in the study was obtained from all subjects. The study protocol was approved by the Medical Ethics Committee of the Campinas State University (UNICAMP), São Paulo, Brazil (N° 393/1997) and Medical Ethics Committee of the University of São Paulo, São Paulo, Brazil (N° 869/06). All patients read and signed the informed consent.

### Exposure

As previously described, there were no systematic fibre measurements in the workplace before 1976. Therefore, a subjective, logarithmic scale was developed to estimate indices of dust exposure for subjects in groups I and II. Fibre measurements performed before the start of the exposure control program were used to establish three levels of workplace dust (mild: from 0.3 to 3 fibres/cubic cm (cc), moderate: from 3 to 30.0 fibres/cc, and severe: more than 30 fibres/cc). These levels were colour coded on a questionnaire as green, white, and red, respectively. Subjects were asked to indicate the colour type and intensity that corresponded best to the average level of dust exposure in their workplaces, from ‘‘no dust at all” (dark green), to ‘‘very dusty” (dark red) [23]. After 1976 (Groups III and IV), measurements of asbestos fibres in the occupational setting were routinely performed. Airborne samples were obtained from different areas of activity in the mining and milling processes, using a constant-flow sampler. Fibre counting was performed according to the National Institute of Occupational Safety and Health standards: a membrane filter was used to collect the fibres and the counting was done with a phase contrast microscope [24]. Briefly, the exposure control program included: the introduction of air filtering, dust collection, humidification, use of individual protection devices, and automatic handling and packing of the milled asbestos. An index of the workers’ asbestos exposure was calculated assuming that the cumulative amount of asbestos exposure in the lungs was proportional to intensity and duration of the inhalation ‘‘load” for each job ever held.

### Spirometry

Spirometric tests were performed with a calibrated pneumotachograph in Multispiro System (Creative Biomedics, San Clement, CA, USA). The subjects completed at least three acceptable maximal forced expiratory manoeuvres and technical procedures followed the recommendations of the American Thoracic Society [25]. The following variables were recorded: forced vital capacity (FVC), forced expiratory volume in one second (FEV1), FEV1/FVC ratio, and forced expiratory flow between 25% and 75% of the FVC (FEF25–75%). Values were compared with those predicted for the adult Brazilian population, taking into account age, gender and height [26].

### Image evaluation

#### Chest radiography (CXR)

Standard high-kilovoltage posteroanterior and lateral CRX were obtained within 1 month of study entry. Three experienced radiologists (15–20 years of experience) graded the radiographs using International Labour Organization [ILO] standards as reference [27]. Recorded values were agreed on by consensus of the readers. Interpretations included determination of category and profusion of pulmonary opacities and width and extent of diaphragmatic, chest wall, and mediastinal pleural plaques. For the present study, pulmonary opacities were reported if the ILO reading was 1/0 or more: occasional disagreements in interstitial and pleural abnormalities reading were sorted out by mode. Median values were considered for the recorded profusion readings.

The readers were blinded to participant identity, physiologic evaluation, and CT results.

#### Thin-section computed tomography

The Thin-section CT images were obtained using an X-vision scanner (Toshiba, Tokyo, Japan). The CT scans consisted of 1-mm or 2-mm collimation at 10 mm intervals performed at full inspiration with the patients in the prone position. All images were reconstructed with a high-spatial-frequency algorithm and photographed for assessment of lung parenchyma (width, 1,200 or 1,500 HU; level, –700 or—800 HU) and pleura and mediastinum (width, 450 or 600 HU; level, 50 or 80 HU). In all patients the images were available and reviewed on hardcopies. Images were randomized and evaluated independently by two thoracic radiologists. The observers were aware that all patients had been exposed to asbestos, but were blinded to patient identification, age, date of CT, smoking history, pulmonary function tests, and radiographic findings. The observers assessed the presence of unilateral or bilateral pulmonary abnormalities with emphasis on the findings described in patients with asbestos exposure [1, 8, 28, 29] which include: thickening of the interlobular septa, intralobular interstitial lines, parenchymal bands, subpleural curvilinear opacities, subpleural dot-like or branching opacities (subpleural nodules), ground-glass opacities, honeycombing, traction bronchiectasis and traction bronchiolectasis. Based on these abnormalities, the readers established, by consensus, whether they were or not “definitively-indicative” of lung fibrosis compatible with asbestosis. Pleural plaques were considered to be present when there was at least one focal pleural abnormality with typical characteristics of a pleural plaque (circumscribed quadrangular pleural elevation with sharp borders and soft tissue density, possibly calcified, in typical posterolateral and anterolateral location).

### Statistical Analysis

All data were collected and presented in contingency tables. Data were tested for normality with the Kolmogorov- Smirnov test. When normality was not observed, non-parametric tests were performed. Categorical variables were tested using the chi-squared or Fisher exact tests. Continuous variables were compared with analysis of variance (ANOVA) or Kruskal-Wallis test, with Bonferroni correction for multiple comparisons. Logistic regression was performed using multivariable analysis to control for confounders. A subgroup of subjects without any Thin-section CT and CXR abnormalities at baseline had been examined at two different time points with CXR and Thin-section CT (N = 301). In these subjects, we performed a longitudinal analysis to assess the incidence of pulmonary disease (asbestosis) and pleural disease (pleural plaques). Incidence rates were calculated. Person-years was defined as time since first exposure to study end date (when outcome was assessed). Incidence rate ratio was calculated using Poisson regression models. P-values less than 0.05 were considered statistically significant and all analyses were performed with STATA v.10 (College Station, Texas, USA).

## Results

Table 1 shows the characteristics of the study population stratified by presence of interstitial abnormalities (IA) or pleural plaques according to the Thin-section CT. Subjects with asbestos-related abnormalities were older with more pack-years of cigarette smoking than subjects without abnormalities (p<0.001). In addition, individuals with IA were exposed during a longer period of time than those with only pleural plaques or normal subjects (p<0.050). Regarding spirometry, those with IA and pleural plaques had lower FVC, FEV1, and FEF25–75% values compared to normal subjects.

**Table 1:** Characteristics of the study population separated by the criterion method (Thin-section CT).

	Normal (N = 1241)	Interstitial Abnormalities (N = 46)	Pleural Plaques (N = 131)	p-value
Gender				0.114
Male	1165 (87.1)	45 (3.3)	128 (9.6)	
Female	76 (95.0)	1 (1.2)	3 (3.8)	
Age, years	57.1 ± 8.7	69.9 ± 8.4	68.1 ± 8.1	a,b
Time of exposure, years	11.2 ± 7.5	14.3 ± 7.5	11.8 ± 8.4	a
Smoking, pack-years	17.9 ± 28.4	37.1 ± 39.6	36.4 ± 39.6	a,b
Smoking				
Never	523 (92.2)	11 (1.9)	33 (5.8)	<0.001
Smoker	159 (82.8)	7 (3.6)	26 (13.6)	
Former Smoker	559 (84.8)	28 (4.2)	72 (11.0)	
Spirometry*				
FVC, L	4.0 ± 0.1	3.1 ± 0.12	3.3 ± 0.1	a,b
FVC, % pred	103.0 ± 0.1	91.6 ± 2.9	92.4 ± 1.8	a,b
FEV_1_, L	3.1 ± 0.1	2.4 ± 0.6	2.3 ± 0.1	a,b
FEV_1_, % pred	100.0 ± 0.1	93.8 ± 3.2	88.6 ± 2.0	b
FEF_25–75%_, L/s	2.9 ± 0.5	2.3 ± 0.2	1.9 ± 0.1	a,b
FEF_25–75%_, % pred	96.9 ± 1.8	90.4 ± 6.0	74.5 ± 3.7	b

Data are frequency (%) or mean ± standard deviation.

*Smoking adjusted mean ± standard deviation.

^a^ p<0.050 comparing Asbestosis vs. Normal.

^b^ p<0.050 comparing Pleural plaques vs. Normal.

Table 2 shows the prevalence of interstitial changes interpreted as consistent with asbestosis and pleural plaques based on the CXR and Thin-section CT. The prevalence of IA was higher on CXR compared to the Thin-section CT. On the other hand, the prevalence of pleural plaques was higher on Thin-section CT. Prevalence of IA and pleural plaques decreased as exposure diminished (i.e., Groups I to IV), regardless of the diagnostic method used.

**Table 2:** Prevalence of normal scan, interstitial abnormalities or pleural plaques according to the specific imaging methods in each exposure group (N = 1418).

	Normal		Interstitial Abnormalities		Pleural Plaques	
	Thin-section CT	CXR	Thin-section CT	CXR	Thin-section CT	CXR
**Group**						
**I**	51 (41.4)	97 (78.8)	12 (9.8)	16 (13.0)	60 (48.8)	10 (8.1)
	(32.6–50.7)	(70.6–85.7)	(5.1–16.4)	(7.6–20.2)	(39.6–57.9)	(3.9–14.4)
**II**	521 (86.8)	554 (92.3)	26 (4.3)	42 (7.0)	53 (8.9)	4 (0.7)
	(83.8–89.4)	(89.9–94.3)	(2.8–6.3)	(5.0–9.3)	(6.7–11.4)	(0.2–1.7)
**III**	45 (95.4)	452 (94.4)	8 (1.7)	23 (4.8)	14 (2.9)	4 (0.8)
	(93.1–97.1)	(91.9–96.2)	(0.7–3.2)	(3.0–7.1)	(1.6–4.8)	(0.2–2.1)
**IV**	212 (98.1)	208 (96.3)	0 (0)	5 (2.3)	4 (1.9)	3 (1.4)
	(95.3–99.5)	(92.8–98.3)		(0.7–5.3)	(0.5–4.6)	(0.3–4.0)

Data are frequency (%).

(95% CI).

Mean ± standard deviation of time from first exposure for all groups was: Group I: 11.9 ± 10.2 years; Group II: 11,1 ± 7.9 years; Group III: 11.4 ± 6.7; Group IV: 11.5 ± 7.0 and it was not different from each other; However, mean ± standard deviation cumulative exposure for all groups was: Group I: 110.9 ± 140.3 fibres-cc-years; Group II: 44.1 ± 49.4 fibres-cc-years; Group III: 7.6 ± 5.4 fibres-cc-years; Group IV: 3.6 ± 4.4 fibres-cc-years. ANOVA test with Bonferroni correction for multiple comparison showed that all values were significantly different from each other (p<0.050).

Table 3 shows the odds ratio and 95% confidence interval (CI) for IA and pleural plaques. An increased odds of developing IA or pleural plaques was associated with increase in age and cigarette smoking. Those who worked in later periods (groups II to IV), when compared to group I, had lower odds of developing IA or pleural plaques. Results were similar when adjusting for smoking status.

**Table 3:** Odds ratio (95% CI) for interstitial abnormalities and pleural plaques according to the specific imaging method (univariate analysis).

	Interstitial Abnormalities		Pleural plaques	
Variable	Thin-section CT	CXR	Thin-section CT	CXR
Age, for 1 year	1.17 (1.13–1.22)	1.08 (1.05–1.10)	1.14 (1.12–1.17)	1.10 (1.05–1.15)
Exposure, for 1 year	1.05 (1.01–1.08)	1.01 (0.98–1.04)	1.01 (0.98–1.03)	0.98 (0.92–1.04)
Smoking, for 1 pack-year	1.01 (1.00–1.02)	1.01 (1.00–1.01)	1.01 (1.00–1.02)	1.01 (1.00–1.02)
Smoking				
Never	1.0	1.0	1.0	1.0
Smoker	2.09 (0.80–5.49)	1.71 (0.87–3.37)	2.59 (1.50–4.46)	2.51 (0.76–8.34)
Former Smoker	2.38 (1.17–4.83)	1.77 (1.08–2.91)	2.38 (1.32–3.13)	1.58 (0.58–4.31)
Group				
I	1.0	1.0	1.0	1.0
II	0.21 (0.10–0.44)	0.49 (0.27–0.89)	0.08 (0.05–0.14)	0.09 (0.03–0.28)
III	0.07 (0.03–0.19)	0.31 (0.16–0.61)	0.03 (0.01–0.05)	0.09 (0.03–0.30)
IV		0.14 (0.05–0.41)	0.02 (0.01–0.05)	0.16 (0.04–0.59)

In the analysis of the smoking-adjusted pulmonary function tests we observed that patients from groups II and III, with pleural plaques evidenced only on Thin-section CT, had consistently lower spirometric values than those deemed as normal by both Thin-section CT and CRX. (Table 4) The very low prevalence of interstitial abnormalities did not allow a statistical analysis of this outcome.

**Table 4:** Smoking-adjusted spirometric variables in patients who presented or not with pleural plaques on CXR and/or Thin-section CT in each group of exposure.

	Group I		Group II		Group III	
	CXR(-)/	CXR(-)/	CXR(-)/	CXR(-)/	CXR(-)/	CXR(-)/
	Thin-section	Thin-section	Thin-section	Thin-section	Thin-section	Thin-section
	CT (-)	CT (+)	CT (-)	CT (+)	CT (-)	CT (+)
	(N = 51)	(N = 51)	(N = 521)	(N = 49)	(N = 454)	(N = 13)
**CVF, L**	3.33 ± 0.12	3.30 ± 0.13	3.95 ± 0.03	3.55 ± 0.05	3.97 ± 0.07	3.18 ± 0.29*
**CVF, %**	98.6 ± 4.4	94.0 ± 2.8	103.9 ± 1.2	90.4 ± 3.5	103.9 ± 1.0	83.3 ± 8.9*
**VEF1, L**	2.66 ± 0.07	2.50 ± 0.15	3.08 ± 0.07	2.65 ± 0.05*	3.10 ± 0.05	2.45 ± 0.40*
**VEF1, %**	97.3 ± 1.16	98.7 ± 5.30	103.2 ± 3.3	87.5 ± 2.5*	102.2 ± 2.77	81.2 ± 13.1*
**FEF**_**25–75**_, **L/s**	2.94 ± 0.22	2.19 ± 0.28*	2.94 ± 0.21	2.19 ± 0.21*	3.01 ± 0.25	2.15 ± 0.48
**FEF25–75, %**	104.5 ± 5.8	87.4 ± 4.9	102.9 ± 8.2	77.8 ± 7.2*	101.7 ± 9.2	72.8 ± 15.9

Data are mean ± standard deviation.

*p<0.050 when comparing those CXR(-) Thin-section CT (+) versus CXR(-) Thin-section CT(-).

### Longitudinal changes in CXT and Thin-section CT

Three-hundred and one individuals (290 males, aged 50.1 ± 8.0 yrs; Group I = 19, Group II = 157, Group III = 109, Group IV = 16) free of asbestos-related abnormalities on both Thin-section CT and CXR in the first evaluation (1997 to 2000) were reevaluated by both imaging methods from 2007 to 2010.

**Fig 1:**
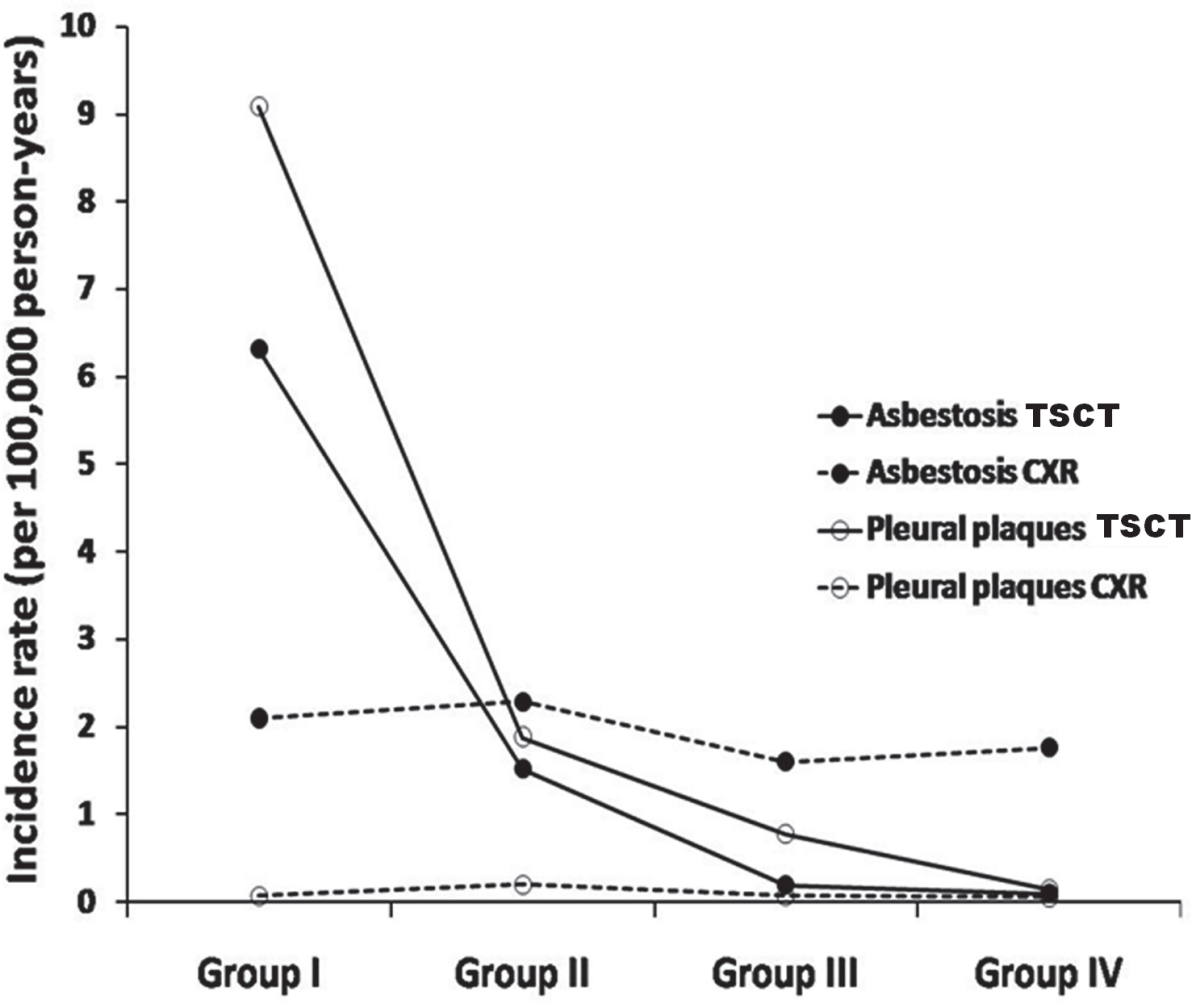
Incidence rates of asbestosis and pleural plaques in groups of decreasing levels of asbestos exposure. Note that the marked reduction in incidence of both asbestosis and pleural plaques from Groups I to IV detected by Thin-section CT (TSCT) was not found in the CXR analysis.

At follow-up, 11 individuals had pulmonary findings consistent with asbestosis on Thin-section CT (overall incidence rate of 1.09 cases of asbestosis per 100,000 person-years). All cases were distributed between groups I (n = 3) and II (n = 8). Eighteen individuals developed pleural plaques evident on Thin-section CT distributed over groups I (n = 5), II (n = 10), and III (n = 3). The overall incidence rate was 1.75 cases of pleural plaques per 100,000 person-years during this time period. When using CXR as the diagnostic method, the overall incidence rate for asbestosis was 1.99 cases per 100,000 person-years (total 20 cases of asbestosis diagnosed by CXR). Cases were distributed across groups I (n = 1), II (n = 12), III (n = 6), and IV (n = 1). There was only 1 case of pleural plaque diagnosed by CXR in group II (incidence rate of 0.20 cases of pleural plaques per 100,000 person-years). There were no incident cases of pleural disease in Group I, III and IV. (Fig. 1) Considering Thin-section CT as the diagnostic method, the incidence rate ratio (IRR) and 95% CI for asbestosis in group II, using group I as reference was 0.24 (0.06–0.90). For pleural plaques, using group I as reference, the IRR for group II was 0.20 (0.07–0.60) and for group III was 0.08 (0.02–0.36). Using CXR as diagnostic method and group I as reference, the IRR for asbestosis in groups II, III and IV were: 1.08 (0.14–8.36); 0.76 (0.09–6.33); and 0.84 (0.05–13.49).

## Discussion

To our knowledge this is the first large study that compared the differences in performance of CXR compared to Thin-section CT in detecting pulmonary and pleural abnormalities in workers with different levels of asbestos exposure in the mining and milling settings. We found that regardless the exposure load, pulmonary abnormalities were more frequently found on CXR than Thin-section CT with the opposite being the case with pleural plaques. Compared to the most exposed group (Group I), reduction of interstitial findings in Groups II to IV was underestimated at CRX compared to Thin-section CT. This was confirmed in a post-hoc longitudinal analysis with a sub-group of patients as only Thin-section CT indicated that the incidence rates of asbestos-related diseases diminished as exposure decreased from Groups I to IV. Altogether these data provide original evidence that the technical limitations of CXR compared to Thin-section CT are of epidemiological relevance as they significantly impair the ability of CRX to detect the expected reduction of abnormalities in contemporary workplaces.

Seminal studies in the 80’s have shown that Thin-section CT is an imaging method particularly suitable for the early detection of anatomical abnormalities associated with asbestos exposure. Several studies consistently demonstrated that Thin-section CT is more sensitive than CRX in demonstrating asbestos related pleural and pulmonary pulmonary abnormalities, even before clinical signs are present [9–11, 13]. The early detection of asbestos-related pulmonary and pleural abnormalities is therefore important for diagnosis, medico-legal questions and to advise the workers to interrupt the exposition by changing their activity. So, Thin-section CT would be the preferred method to evaluate workers and ex-workers at risk [1, 2, 8, 9, 30, 31]. Compared to radiography, however, Thin-section CT is not only a costlier and more complex procedure but it is also associated with greater radiation exposure [32]. Therefore, the precise role of Thin-section CT in large-scale evaluations of occupationally-exposed subjects remains uncertain.

The present study demonstrates that regardless the pre-test risk of disease (as indicated by differences in cumulative exposure and time to first exposure) Thin-section CT is superior to radiography in the detection of pleural plaques and avoids the false positive interpretations of pulmonary disease by the CXR. Because of the misclassification of both abnormalities on the CXR the decreased prevalence of pleural and pulmonary changes with decreasing exposure to asbestos (Groups I to IV) was less discernible on radiography than on Thin-section CT (Table 2). Considering just the Thin-section CT evaluation, the higher prevalence of pleural plaques and pulmonary changes observed in Group I, was mostly related to a higher cumulative exposure, (even with the same time from the first exposure) and to exposition to both chrysotile and amphiboles, compared to other groups. These findings are likely to be applicable for most of the workplace settings as the more recent studies indicate that disease prevalence is lower than reported in older cohorts [5, 6, 31, 33]. Our data also suggest that measures to reduce asbestos exposure can be highly effective, being accompanied by decreases in disease prevalence and incidence (Table 2). From an epidemiological point-of-view, therefore, the present data indicate that Thin-section CT is superior to radiography in adequately quantifying the reduction in the frequency of interstitial abnormalities with decreased levels of exposure. However, it should be pointed out that the choice between the two methods to screen workers at low risk should also take into consideration economical (cost/benefit ratio) and operational issues which were not addressed in the present study [31, 33].

There are two additional findings of the present study that deserve further consideration. Firstly, it was rather surprising that the prevalence of IA was lower at Thin-section CT than CXR (Table 2) and the latter method indicated a greater number of incident cases. In other words, the small opacities described on the CXR were not confirmed by the Thin-section CT scans. Disagreements between CXR and Thin-section CT based diagnoses have been described previously in the literature in other pulmonary and pleural diseases [17–19].

Secondly, smoking-adjusted lung function parameters were consistently lower in patients in whom pleural plaques were found only at Thin-section CT (Table 4). In these subjects, reductions in FEV1 and mid-expiratory flows are generally proportional to decreases in FVC thereby indicating a trend to increased elastic recoil due to lower lung volumes, i.e., a “restrictive” pattern of ventilatory dysfunction. These findings are consistent with recent evidence that pleural abnormalities, even if not extensive, are not functionally innocuous to these patients and should be clinically valued [34].

As previously described in this disease population, most subjects were smokers or ex-smokers (Table 1). Smoking retards the lung clearance of asbestos fibres and may contribute to the severity and progression of asbestosis [35, 36]. On the other hand, smoking increased all emphysema signs and leads to bronchial wall thickening but was negatively associated to CT signs of septal lines and parenchymal bands [36]. However, a previous study from our group showed that the occurrence of emphysema on Thin-section CT was less than 5% in most patients with asbestos related disease, so we do not believe that such abnormalities have hampered the interpretative analysis of our data [19]. A review article revealed that despite the higher cost radiation dose, Thin-section CT is useful in the investigation of chronic diffuse pulmonary disease with normal radiographs, with approximately 10–15% of patients having a biopsy confirming infiltrative pulmonary disease, 30–50% having bronchiectasis and 20–60% having emphysema [32].

Our longitudinal analysis confirmed the findings from our cross-sectional study. After 10 years of follow-up, we also observed a decrease in the incidence of abnormalities as the level of exposure decreased. Interestingly, Thin-section CT was the method that best identified differences between the groups (Fig. 1). Other studies using CXR as a diagnostic tool, showed reduction of pulmonary abnormalities with decreased cumulative asbestos exposure [14, 37].

The present study has some relevant limitations. Survival bias might have influenced our results; however, this is unlikely to represent a serious shortcoming in this sample of subjects with mild disease. Absence of histopathologic proof of asbestosis might be viewed as a study limitation; however, the current consensus is that there is no justification for surgical lung biopsy in patients who are suspected of having asbestosis based on clinical data and an appropriate history of asbestos exposure. The present findings should not be extrapolated for groups presenting with extensive pulmonary and/or pleural abnormalities where the differences in CXR and Thin-section CT are likely to be less relevant in practical terms.

We conclude that CXR compared to Thin-section CT was associated with false-positives for IA and false-negatives for pleural plaques in miners and millers with decreasing levels of asbestos exposure. This led to substantial misinformation of the effects of lowering exposure to asbestos fibres in the occurrence of asbestos-related abnormalities over time.

Our data support the notion that Thin-section CT should be considered, instead of the CXR, in the longitudinal evaluation of subjects exposed to low dose asbestos fibres.
